# Efficient algorithms for reconstructing gene content by co-evolution

**DOI:** 10.1186/1471-2105-12-S9-S12

**Published:** 2011-10-05

**Authors:** Hadas Birin, Tamir Tuller

**Affiliations:** 1 School of Computer Science, Tel Aviv University, Israel; 2 Department of Biomedical Engineering, Faculty of Engineering, Tel Aviv University, Tel Aviv, Israel

## Abstract

**Background:**

In a previous study we demonstrated that co-evolutionary information can be utilized for improving the accuracy of ancestral gene content reconstruction. To this end, we defined a new computational problem, the Ancestral Co-Evolutionary (ACE) problem, and developed algorithms for solving it.

**Results:**

In the current paper we generalize our previous study in various ways. First, we describe new efficient computational approaches for solving the ACE problem. The new approaches are based on reductions to classical methods such as linear programming relaxation, quadratic programming, and min-cut. Second, we report new computational hardness results related to the ACE, including practical cases where it can be solved in polynomial time.

Third, we generalize the ACE problem and demonstrate how our approach can be used for inferring parts of the genomes of *non-ancestral* organisms. To this end, we describe a heuristic for finding the portion of the genome ('dominant set’) that can be used to reconstruct the rest of the genome with the lowest error rate. This heuristic utilizes both evolutionary information and co-evolutionary information.

We implemented these algorithms on a large input of the ACE problem (95 unicellular organisms, 4,873 protein families, and 10, 576 of co-evolutionary relations), demonstrating that some of these algorithms can outperform the algorithm used in our previous study. In addition, we show that based on our approach a ’dominant set’ cab be used reconstruct a major fraction of a genome (up to 79%) with relatively low error-rate (*e.g.* 0.11). We find that the ’dominant set’ tends to include metabolic and regulatory genes, with high evolutionary rate, and low protein abundance and number of protein-protein interactions.

**Conclusions:**

The *ACE* problem can be efficiently extended for inferring the genomes of organisms that exist today. In addition, it may be solved in polynomial time in many practical cases. Metabolic and regulatory genes were found to be the most important groups of genes necessary for reconstructing gene content of an organism based on other related genomes.

## Introduction

Reconstruction of ancestral genomic sequences is a classical problem in molecular evolution. The first algorithm for reconstructing ancestral genomic sequences was suggested around 40 years ago by Fitch [1]. This algorithm was based on the Maximum Parsimony (MP) criteria and was designed for sequences with a binary alphabet. A few years later the algorithm was generalized by Sankoff, for inputs with non-binary alphabets and multiple edge weights [2]. More recently, similar approaches for optimizing the maximum likelihood score (ML; instead of maximum parsimony) emerged [3-8].

Reconstruction of ancestral genomic sequences was employed in many biological and bioinformatical studies in recent years. Specifically, it was used for studying various evolutionary questions [9-18]), for aligning genomic sequences [19], and for inferring ancestral SNPs [20].

In practice, the solution space of the ancestral sequences reconstructing problem, tends to be populated with a large number of local and global maxima, obscuring algorithm accuracy. Thus, the ancestral sequences inferred by the conventional approaches tend to have a relatively large number of errors. Based on the fact that functionally and physically interacting proteins tend to co-evolve [21-25], we have recently suggested the Ancestral Co-Evolver approach, for improving the accuracy of reconstructed ancestral genomes [26,27]. Our approach was based on utilizing information embedded in the co-evolution of functionally/physically interacting proteins.

The current study includes novel algorithms for the ACE problem. In addition, we generalize our previous approach showing that co-evolution is not only an important statistical force that can be employed to infer ancestral sequences, but it can also be used for inferring the genomes of organisms existing today (*i.e.* the leaves of the evolutionary tree). Such an approach can be utilized for the analysis of metagenomic data (see, for example, [28]). Furthermore a generalization of this approach can be used for inferring biological networks (*e.g.* protein-protein interactions and metabolic networks [29,30]). As we demonstrate in this paper, this approach is also a useful tool for studying genomic and molecular evolutionary.

The rest of the paper is organized as follows. In subsection ’Definitions and Preliminaries’, we define the notations and computational problems studied in the paper. In subsection ’Some Computational Issues’, we deal with the computational hardness of the ACE problem. We show that in many practical cases it can be solved in polynomial time. In subsection ’Methods and Algorithms’, we describe the biological data used in this study, and a new set of algorithms for solving the ACE problem. In addition, we describe a new approach for detecting a part of the genome, which can then be used for inferring the remaining gene content, with the lowest error rate. In the last three subsections, we demonstrate the ACE algorithms’ performance, by analyzing a large dataset (an evolutionary tree, genomes and co-evolutionary relations) corresponding to 95 unicellular organisms [26], and discuss their features. The section ’Conclusions’ includes concluding remarks and a discussion.

## Results and discussion

### Definitions and preliminaries

For simplicity, we assume a binary alphabet. However, all the results here can be easily generalized to models with more than two characters (see examples in [26]). Each genome is represented by a binary sequence corresponding to the states of all the proteins in the genome. If the value of the *i*-th bit of the sequence is ’1’, it means that the *i*-th protein is encoded in the genome; if the *i*-th bit of the sequence is ’0’, then the *i*-th protein is not encoded in the genome. As we explain later, there may also be bits with unknown values (i.e. it is not known if the protein appears in the genome or not); we use the label ’?’ for such cases. In the current study, our aim is to in addition infer these missing values.

In this work, neighbor sites in the input sequences evolve independently, when they do not have a known co-evolutionary relation. Thus, the basic components in the model and algorithms are *single* characters. Our goal is to reconstruct the ancestral states and missing states at the leaves, for a set of organisms  of size . A *phylogenetic tree* is a rooted binary tree *T* = (V(*T*), E(*T*)) with a *leaf labeling* function *λ*, where V(*T*) is the set of vertices and E(*T*) the set of edges.

In our context, a weight table is attributed to each edge (*u*, *v*) = *e* ∈ E(*T*). This *weight table* includes a weight (a positive real number), for each pair of labels of two vertices (*u*, *v*) = *e*.

In this work, we assume that each node in a phylogenetic tree corresponds to a different organism. The leaves in a phylogenetic tree correspond to organisms existing today , while the internal nodes correspond to organisms that have become extinct . Thus, we can name each node after its corresponding organism. Let *O_T_*(⋅) denote a function that returns the index of the organisms corresponding to each node in *T*, *i.e.* for every *v* ∈ *V*(*T*), *O_T_*(*v*) is the index of the organism (from ) corresponding to *v.*

The *leaf labeling* function is a bijection between the leaf set L(*T*) and the set of genomic sequences (or subsequences) corresponding to the organisms that exist today, . In our binary case, each label is a binary sequence with missing entries and all the sequences have the same length. As with conventional ML/MP, we assume an *i.i.d.* case, where different characters in a sequence evolve independently, thus we can describe an algorithm for sequences of length one (*i.e.* each sequence is ′1′, ′0′, or ′?′).

A *full labeling* of a phylogeny , is a labeling of *all* the nodes of the tree such that the labels of the leaves that are not missing are the same as the non-missing values of *λ*, *i.e.*, for all cases that are not missing values, . In the current study, we solve the gene content inference problem; where each character in a label corresponds to a protein in a genome. As previously stated, if the value of a character is ′1′ it means that the protein is coded in the genome and if it is ′0′ it means that the protein is not coded in the genome.

**Figure 1:**
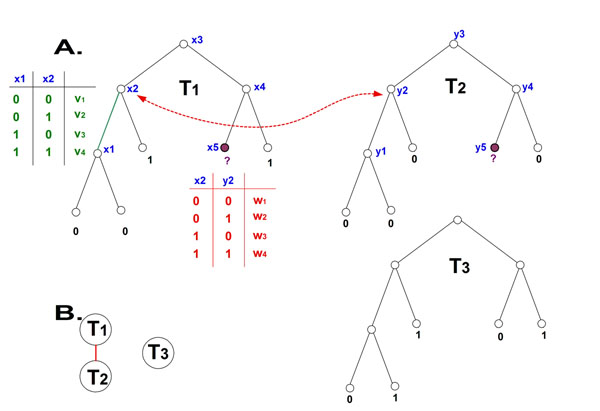
**The Ancestral Co-Evolution*** A.* A simple example of a *co-evolving forest* with three trees (each tree corresponds to a different gene family), and one *co-evolution edge* connecting node *x*_2_ in tree *T*_1_ and node *y*_2_ in tree *T*_2_; the weight table corresponding to this co-evolution edge is in red. The weight table corresponding to the tree edge (*x*_1_, *x*_2_) in *T*_1_ is in green. The values at the leaf *x*_5_ in tree *T*_1_ and the leaf *y*_5_ in tree *T*_2_ are missing. *B.* The *co-evolutionary graph* corresponding to the *co-evolving forest* in *A.*

A *co-evolving forest F* =(*S_F_* = {*T*_1_, *T*_2_, *…*}, *E_c_*(*S_F_*)) is a set of *phylogenetic trees*, *S_F_*, with *identical* topology that correspond to the same organisms [*i.e.* each tree has the same *O*(·)], and an additional set of edges, *E_c_*(*S_F_*), that connect pairs of nodes in *different* trees. This set of edges represents the co-evolutionary relations between pairs of protein families. Edges in *E_c_*(*S_F_*) must connect pairs of nodes that correspond to the same organism (*i. e.* (*v*, *u*) ∈ *E_c_*(*S_F_*), *v* ∈ *V*(*T*_1_), *u* ∈ *V*(*T*_2_) ⇒ *O*_*T*_1__(*v*) = *O*_*T*_2__(*u*); Figure 1); we call such pairs of nodes *legal co-evolutionary pairs.*

The edges in *E_c_*(*S_F_*) are named *co-evolution edges*, while edges that constitute the evolutionary trees are named *tree edges.* For example, Figure 1A. includes a *co-evolving forest* with two trees (the *co-evolution edges* are dashed with arrows, while the *tree edges* are continuous). In this work we assume that new *co-evolutionary edges* do not appear/disappear during evolution. Namely, we assume that if there is a *co-evolutionary edge* between a *legal co-evolutionary pair* of nodes in two trees, then all the *legal co-evolutionary pairs* of nodes in the two trees are connected by *co-evolutionary edges.* In this study, we also assume that there is no change in the co-evolutionary weight table, across *legal co-evolutionary pairs* of nodes corresponding to a pair of phylogenetic trees. However, with suitable biological support/data, the co-evolutionary weight tables may differ across a pair of evolutionary trees (reflecting changes in co-evolutionary relations across evolution). Thus, the parsimony score in the case of the ACE problem can capture the evolutionary events of proteins, while considering our belief regarding the dependencies between pairs of proteins.

A *full labeling* of a *co-evolving forest* is a full labeling, , of all the nodes of the phylogenetic trees in *S_F_*, including the missing values at their leaves. The roots of a *co-evolving forest* are the set of roots of the *phylogenetic trees* in the *co-evolving forest*.

As mentioned, a *co-evolving forest* also includes a weight table for each *co-evolution edge* and each *tree edge*. These weight tables are cost functions, which return a real positive number for each pair of labels at the two ends of the edge. In the case of *tree edges*, these weights reflect the probability of a mutation along the edge. In the case of *co-evolution edges*, these weights reflect the distribution of mutual occurrence of the labels of the nodes at the ends of the edge.

This leads us to the formal definition of the problem we are concerned with, the *Ancestral Co-Evolution* (ACE) problem with missing variables at the leaves, which is a *generalization* of the problem defined in [27]:

**Problem 1** Ancestral Co-Evolution (*ACE*)

**Input:** A *co-evolving forest*, *F* = (*S_F_*, *E_c_*(*S_F_*)) (possibly with missing labels at the leaves), and a real number, *B*.

**Question:** Are there labels for the internal nodes of all the trees in the *co-evolving forest*, and the missing values at the leaves, such that the sum of the corresponding weights along all the tree edges and the *co-evolution* edges is less than *B*?

Note that in general, it is not necessarily required that the solution for each tree *separately*, will be the most parsimonious. The minimal sum of edge weights corresponding to a *co-evolving forest*, *F* (Problem 1) is denoted the *cost* of *F*. A *co-evolutionary graph* is an undirected graph, which describes the *co-evolution* edges in the *co-evolving forest*. In such a graph, each node corresponds to a tree in the *co-evolving forest*, and two nodes are connected by an edge, if there is at least one co-evolution edge between their corresponding trees. A connected component in the *co-evolving forest* is a sub-set of trees, such that their corresponding nodes in the co-evolutionary graph induce a connected component (see an example in Figure 1B.).

It is easy to see (more details in [26]) that if the optimization criterion is maximum likelihood (see, for example, [4]) for *i.i.d* models such as Jukes Cantor (JC) [31], Neyman [32], or the model of Yang *et al.*[*33*], the problem can be formalized as a maximum parsimony problem with a non-binary alphabet and multiple edge weights [2]. Thus, the *Ancestral co-evolution* problem without *co-evolution edges* (|*E_c_*(*S_F_*)| = 0), can describe a Maximum Likelihood (ML) problem.

**Figure 2:**
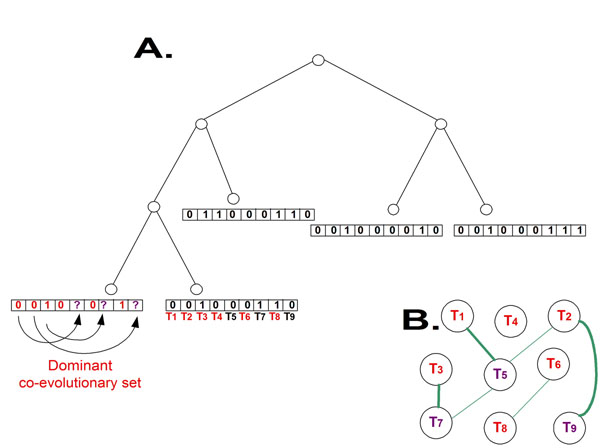
**The Dominant Co-Evolutionary Set (DCES) problem ***A.* The input to the DCES problem is a phylogenetic forest and a target genome (red) corresponding to a certain leaf in all the phylogenetic trees (the leftmost leaf in this example). The goal is to find a subset of proteins in the target genome such that given this subset of proteins (and the phylogenetic forest), it will be possible to infer the rest of the genome; in this example, proteins from gene families *T*_1_, *T*_2_, *T*_3_, *T*_4_, *T*_6_, *T*_8_ are used to infer the rest of the target genome (the proteins marked with *^′^*?*^′^*; proteins *T*_5_, *T*_7_, *T*_9_ in the example). *B.* The problem can be reduced to a version of the dominant set problem [34] (see details in section ’Algorithm for the Dominant co-evolutionary set problem’). In the reduction we build a graph that includes a node for each gene family and pairs of gene families are connected with edges if they have a strong co-evolutionary relation; we want to find a dominant set in this graph.

In this paper, we also study the problem of finding a sub-set of the genes in a genome (one of the leaves in some of the phylogenetic trees), such that it can be used for reconstructing the rest of the gene content of this genome with minimal error-rate, based on the information embedded in the co-evolutionary forest (see Figure 2). We named this problem the *Dominant Co-Evolutionary Set* (DCES) problem (more details in section ’Algorithm for the *Dominant co-evolutionary set* problem’).

### Some computational issues

It has been shown that the ACE problem is NP-hard by a reduction from the MAX-2SAT problem [27]. In this section, we describe another simple reduction from/to the ACE problem, and will use it to prove that the hardness of the problem is related to anti-correlative weight tables. In many practical cases the anti-correlative relations are rare; thus, the ACE problem can be solved in polynomial time.

Let (*a*, *b*, *c*, *d*) denote the notation for a weight table (either a weight table of tree edges or of co-evolutionary edges, see Figure 1), where the costs *a*, *b*, *c*, *d* are for the labels 00, 01, 10, 11 respectively at the ends of the edge. Assume that the analyzed co-evolutionary forest includes two types of edges: 1) green (”good”) edges of the form (0,1,1, 0) corresponding to a positive correlation between the two proteins along the tree edges (*i.e.* the two proteins tend to appear/disappear in the same organism); 2) red (”bad”) edges of the form (1, 0, 0, 1), corresponding to a negative correlation between the two proteins (*i.e.* when one of the proteins appears in a genome, the second usually does not). Note that these two types of edges are the most informative ones (*e.g.* in terms of entropy). Indeed, such edge weights have been included in previous studies. For example, the classical algorithm of Fitch [1] considers only green edges.

If all the edges are green, the problem becomes a Min-Cut (defined below), which is polynomially solvable. Thus, if all the weight tables are of the form (0, 1, 1, 0) (as in [1]), *any* topology of the co-evolutionary forest of the ACE problem, can be solved in polynomial time.

**Problem 2** Min-weighted Cut

**Input:** A weighted graph *G* = (*V*, *E*, *W*(*E*)).

**Solution:** A cut *C* = (*S*, *T*) which is a partition of *V* of the graph *G.*

**Objective:** Minimize the total weight of all edges that are in the set {(*u*, *v*) ∈ *E*|*u* ∈ *S*, *v* ∈ *T*}.

In the following lemma we formally show a reduction from the ACE problem to the min-cut problem, for the case where all the weight tables are of the form (0, 1, 1, 0). A similar reduction can be employed for reducing min-cut to ACE.

**Lemma 1** The ACE problem with only weight tables of the form (0, 1, 1, 0) can be reduced to the min-cut problem.

**Proof** Given a phylogenetic forest as an input to the ACE, problem the instance of the min-cut problem includes a graph *G* = (*V*, *E*), that is reconstructed as follows: *V* is the set of nodes of the phylogenetic forest (*i.e.* the nodes of all the phylogenetic trees); *E* is the set of edges in the co-evolutionary forest (both tree edges and co-evolutionary edges).

Now, we will show that there is a cut of size |*C*| in *G* iff the score of the ACE problem is |*C*|.

⇒ Suppose that there is a minimal cut *C* = (*S*, *T*), such that the size of the cut is |*C*|. In the ACE problem, label all the nodes in *S* with ’1’, and all the nodes in *T* with ’0’. The edges (tree edges or co-evolutionary edges), which increase the score of the ACE problem include only the edges of the cut (other edges have two identical labels at their ends). By the definition of the weight table, each of these edges increases the ACE score by 1. Thus, there exists a labeling for the ACE problem with score |*C*|.

⇐ Suppose that there is a labeling for the ACE problem with score |*C*|. In the min-cut problem select all the nodes that have the label ’1’ to be in *S*, and all the nodes with label ’0’ to be in *T.* By the definition of the weight tables, only edges with non-identical labels at their ends contribute 1 to the ACE score, and each of these edges is in the cut. Thus, the size of the cut is |*C*|.

□

However, if all tables are of the form (1, 0, 0, 1), the problem becomes Min-UnCut, which is NP-hard (like Max-Cut) [34].

**Problem 3** Min-weighted UnCut

**Input:** A weighted graph *G* = (*V*, *E*, *W*(*E*)).

**Solution:** A cut *C* = (*S*, *T*) which is a partition of *V* of a graph *G.*

**Objective:** Minimize the total weight of all edges that are *not* in the cut (*i.e.* minimize the set {(*u*, *v*) ∈ *E*|((*u*, *v*) ∈ *S*) ∨ ((*v*, *u*) ∈ *T*)}.

In the following lemma we formally show a reduction from the ACE problem, to the min-Uncut problem, for the case that all the weight tables are of the form (1, 0, 0, 1). A similar reduction can be applied for reducing min-Uncut to ACE.

**Lemma 1** The ACE problem with all the weight tables of the form (1, 0, 0, 1) can be reduce to the min-UnCut problem.

**Proof** Given a phylogenetic forest as an input to the ACE problem, the instance of the min-Uncut problem includes a graph, *G* = (*V*, *E*) that is reconstructed as follows: *V* is the set of nodes of the phylogenetic forest (the nodes of all the phylogenetic trees); *E* is the set of edges in the co-evolutionary forest (tree edges and co-evolutionary edges).

⇒ Suppose that there is a minimal UnCut *C* = (*S*, *T*) such that the size of the UnCut is |*C*|. In the ACE problem label all the nodes in *S* with ’1’, and all the nodes in *T* with ’0’. The edges (tree edges or co-evolutionary edges) that increase the score of the ACE problem, are only the edges that are *not* in the cut (other edges do not have two identical labels at their ends and according to the weight table the weight of such edges is 0). By the definition of the weight table, each of these edges increases the ACE score by 1. Thus, there is a labeling for the ACE problem with score |*C*|.

⇐ Suppose that there is a labeling for the ACE problem with score |*C*|. In the min-UnCut problem, select all the nodes that have the label ’1’ to be in *S*, and all the nodes with label ’0’ to be in *T.* By the definition of the weight tables, only edges with two identical labels at their ends contribute 1 to the ACE score, and each of these edges are is *not* in the cut. Thus, the size of the UnCut is |*C*|.

*□*

Let *tu* denote the upper bound on the number of possible assignments to the internal nodes, and the missing values at the leaves of a single tree, in the co-evolutionary forest *S_F_* (*i.e.* in a co-evolutionary forest in which the evolutionary trees have *n* nodes *tu* = 2*^n^*). Let *T_MinCut_*(*S_F_*) denote the (polynomial) computational time it takes to solve the min-cut problem corresponding to the co-evolutionary forest *S_F_.* It is easy to see that if the co-evolutionary forest includes *r* red edges, the optimal assignment can be found in *O*(*tu*^2^*^·r^ · T_MinCut_*(*S_F_*)) = *O*(2^2^*^·r·n^ · T_MinCut_*(*S_F_*)), by implementing the min-cut algorithm on all possible assignments to the evolutionary trees at the ends of the red edges. Thus, this is a Fixed-Parameter Tractable (FPT) algorithm with a running time that is exponential with the number of red edges and the size of the evolutionary trees. For example, if we consider *only* the co-evolutionary information (see, for example, [26]), an input with *r* red edges can be solved in *O*(2*^r^ · T_MinCut_*(*S_F_*)) time complexity.

Finally, it is easy to see that the results reported in this section can be generalized to the case where the edge tables include *α* instead of 1 and *β* instead of 0, and *β* is small relatively to *α* (*i.e. β* <*α*/|*E*(*S_F_*)|*;* and *E*(*S_F_*) is the set of edges in the phylogenetic forest).

### Algorithms

This section includes a few algorithmic approaches for inferring genomic sequences by co-evolution. The first approach was suggested in our previous paper, whilst the rest are novel.

#### A FPT algorithm and approximation heuristics

Here we describe very briefly the FPT algorithm and corresponding approximation heuristics that were described in [27]. This heuristic approach has 3 major steps: 1) clustering/dividing the co-evolutionary forest to small enough sub-forests (with relatively many co-evolutionary relations among phylogenetic trees from the same cluster/sub-forests); 2) Using a dynamic programming algorithm (a version of the Sankoff algorithm [2]) for finding exact solutions for each of these sub-forests; 3) Improving the solution found in step 2) greedily. The algorithm that is employed in step 2) finds the exact optimal solution for the ACE problem, but its running time is exponential with the size of the largest connected component in the co-evolutionary graph.

### A Quadratic and Linear Programming

**Figure 3:**
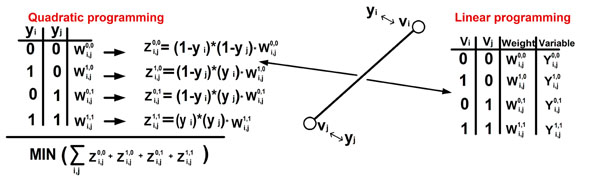
**Linear and Quadratic Programming for solving the ACE problem.** Linear and Quadratic Programming for solving the ACE problem.

In this subsection we demonstrate how the ACE problem can be formulated as a Quadratic Programming (QP), and a Linear Programming (LP) problem. To this end we define several variables that will be used in these formulations. For each node *v_i_* in the co-evolutionary forest (*i.e.* one of the nodes in the phylogenetic trees that are in the phylogenetic forest), we define a variable *y_i_*; In addition, for each edge (*v_i_*, *v_j_*) in the co-evolutionary forest, we define four variables, which we name *edge variables*, one for each possible assignment of the ends of the edge . Let  denote the four weights in the weight table of the edge (*v_i_*, *v_j_*) (see Figure 3). We will start with a definition of Quadratic Programming (QP). Let *x* ∈ *R_n_* denote a set of *n* variables; let *c*, *x_L_*, *x_U_* ∈ *R_n_* denote vectors of real numbers; let *A* ∈ *R_m_*_∗_*_n_* be a matrix of real numbers; *F* is a symmetric positive-definite matrix; let *b_L_*, *b_u_* ∈ *R_m_* be vectors of *m* real numbers. The general formulation of a Quadratic Programming is as follows:

*min_x_f*(*x*) = 0.5 · *x^t^ · F · x* + *c^t^ · x*

such that:

(1) *x_L_* ≤ *x* ≤ *x_U_*

(2) *b_L_* ≤ *Ax* ≤ *b_U_*

In the case of *Integer* Quadratic Programming (IQP) or integer programming, all the variables are integers (*i.e.* either ’0’or ’1’).

The ACE problem can be easily defined as an IQP problem (see Figure 3). In this case we consider the *y_i_* variables defined above. These variables are 0 ≤ *y_i_* ≤ 1 in the case of QP and *y_i_* = {0, 1} in the case of IQP. Based on these variables and the weights in the weight tables, we define for each edge (*v_i_*, *v_j_*) four terms:  (details in Figure 3; in the case of *y_i_* = {0, 1} only one of these terms is larger than zero). The (Quadratic) optimization function is . In the case of *y_i_* = {0, 1}, for each edge only one of the terms in the weight tables is larger than zero.

As we show in the next section, solving IQP for large inputs of the ACE is time consuming, and not practical for large inputs. However, for small inputs, such an approach may be useful.

In the rest, of this subsection we will show how to formulate a Linear Programming (LP) relaxation or an Integer Programming (IP) of the ACE problem. The general formulation of a linear programming is as follows:

*min_x_f*(*x*) = *c^t^ · x*

such that:

(1) *x_L_* ≤ *x* ≤ *x_U_*

(2) *b_L_* ≤ *Ax* ≤ *b_U_*

The following is the reduction to a LP relaxation of the ACE problem (Figure 3):

A. The variables:

(1)  are edge variables, such that each of them hold a value stating whether this corresponding assignment (*i.e.* the labeling of the two ends of the edge) was chosen for this edge, (in the integer programming case for each *i*, *j* only one of the terms is 1 and the rest are 0).

(2) The *y_i_* variables. Each of them should hold the value stating the appropriate assignment for node *i* in the co-evolutionary forest (0 or 1 in the case of integer programming).

B. The target function:

*x* is a vector that includes all the variables mentioned in *A.* The costs that are related to the edge variable (*i*, *j*) are the corresponding weights in the weight table (Figure 3); *i.e.*. The cost corresponding to all the variables *y_i_* is 0.

C. Constraints on the variables:

(1) All variables must receive a value from [0, 1], *i.e.*:

0 ≤ *y_i_* ≤ 1

(2) Every edge must get exactly one assignment, *i.e.*:

(3) Every node must have a consistent assignment across all edges touching it. Thus, for every edge (*i*, *j*) touching node *i*, it must hold that . Thus, in the integer case either *y_i_* = 0 or *y_i_* = 1. If *y_i_* = 0 every edge that includes *i* gets an assignment where node *i* is assigned with 0; similarly, for *y_i_* = 1 the edges that include are assigned such that node *i* is equal to 1.

D. The Size of the problem:

Let *E*(*S_F_*) and *V*(*S_F_*) denote the total number of edges and nodes in the co-evolutionary forest respectively. The number of variables in the LP: 4 · |*E*(*S_F_*)| + |*V*(*S_F_*)|; The number of constraints in the LP: 3 · |*E*(*S_F_*)|.

Thus, with the reductions described in this subsection, packages that solve IQP, QP, LP, or IP can be used for solving the ACE problem.

### A Min-Cut based heuristic

As we mentioned in section ’Some Computational Issues’, when the input includes only green edges it becomes a Min-Cut and can be solved in polynomial time.

Thus, a possible heuristics based on this phenomenon includes the following steps:

1) Consider only the ”good” edges (round the weight table of these edges to be of the type (0, *A*, *A*, 0)) and find the mean cut solution for these edges. We implemented the min-cut algorithm of Stoer-Wagner [35].

2) Start with the min-cut solution found in 1) and run a greedy algorithm based on the *entire* set of edges (see [27] ).

If the number of ”bad” edges is small one can implement an FPT that is exponential with the number of ”bad” edges ( for each assignment of the bad edges, run max-cut to find the assignment for the ”good” edges; as was mentioned in section ’Some Computational Issues’).

### Algorithm for the *Dominant co-evolutionary set* problem

In this subsection we describe a heuristic for solving the *Dominant Co-Evolutionary Set* (*DCES*) problem. The aim is to find a set of gene families (for example, COGs [36]), that we name a ’dominant set’ (*DS*), such that in a certain organism (*i.e.* a target genome) the proteins corresponding to this *DS* can be used for reconstructing the *rest* of the proteins in the genome, with an error-rate lower than a certain threshold. The missing proteins in the genome are reconstructed based on the *DS*, co-evolution and evolutionary information.

The central idea of our heuristic is a reduction of the DCES problem to a *version* of the dominant set problem which is described below. The following is the formal definition of the dominant set problem.

**Problem 4** Dominant set

**Input:** A graph *G* = (*V*, *E*, *W*(*E*)).

**Solution:** A subset *D* ∈ *V* such that every vertex not in *D* is joined to at least one member of *D* by some edge.

**Objective:** Minimize the size of *D.*

**Figure 4:**
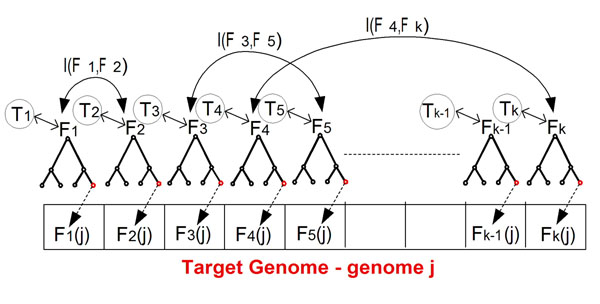
**The reduction used for solving the DCES problem.** The reduction used for solving the DCES problem.

Let *W*_1_ and *W*_2_ denote two thresholds. A gene family is a specific phylogenetic tree in the co-evolutionary forest. The relevant values corresponding to such a gene family in the current context are the labels at the *leaves* of the gene family tree. Given an input co-evolutionary forest and a target genome *j*, we perform the following steps (see also figure 4):

1. Set a variable *F_i_* for each gene family in the co-evolutionary forest, and generate a graph with a node for each *F_i_*. For each *F_i_* there is a related binary vector corresponding to the values of the gene family in the different organisms. *F_i_*(*j*) = 1 designates that the gene family is encoded in genome *j*, *F_i_*(*j*) = 0 designates that the gene family is not encoded in genome *j.*

2. Set a variable *T_i_* for each protein in the *target genome* (*e.g.* genome *j*). This variable represents how well we can infer the value of *F_i_*(*j*) based on the tree structure, and the labels of the other leaves of the tree (*i.e.* the values of the gene family *F_i_* in the rest of the organisms).

3. Let *MP*(*Ti*|*F_i_*(*j*) = 0), *MP*(*Ti*|*F_i_*(*j*) = 1) denote the parsimony score of the evolutionary tree corresponding to the gene family *F_i_*, when setting the values of this gene family in genome *j* (the target genome) to be *F_i_*(*j*) = 0 and *F_i_*(*j*) = 1 respectively. Connect each *T_i_* as a node to the corresponding *F_i_* node with an edge weight *W*(*Ti*) = |*MP*(*Ti*|*F_i_*(*j*) = 0) *– MP*(*Ti*|*F_i_*(*j*) = 1)|/(*min*{*MP*(*Ti*|*F_i_*(*j*) = 1), *MP*(*Ti*|*F_i_*(*j*) = 0)}). Roughly speaking a larger *W*(*Ti*) signifies that with higher probability we can reconstruct *F_i_*(*j*) based on the evolutionary tree of *F_i_*.

4. Based on the binary vector related to each *F_i_*, compute for each *F_i_* its empirical entropy, *H*(*F_i_*)*;* compute for each pairs of variables *F_i_*, *F_l_* the empirical mutual information (*I*(*F_i_*, *F_l_*)). Connect each pair of variables *F_i_*, *F_l_* by an edge with weight *I*(*F_i_*, *F_l_*).

5. The result of the previous steps is a weighted graph that represents the relations between all the *F_i_* and *T_i_* variables defined above (see figure 4). We want to find a minimal set (DS) of *F_i_* variables such that each variable, *F_i_ not* in the DS, either has a strong connection to its *T_i_* variable (*i.e.* its inference strength, based on the evolutionary tree as the edge weight to the *T_i_* variable, is above *W*_2_) or/and it has strong connections to the other nodes in the DS (*i.e.* it can be inferred based on the co-evolutionary information – there is a set of nodes *F_k_*_1_ , *F_kn_*, in the DS such that [*H*(*F_i_*) – (∑*_kj_*_∈_*_DS_ I*(*F_i_*, *F_kj_*))] <*W*_1_).

6. All the nodes *F_i_* that have weak co-evolutionary relations *H*(*F_i_*) – (∑*_kj_I*(*F_i_*, *F_kj_*) >*W*_1_ and their connection to the tree (*T_i_*) is weak <*W*_2_ should be in the resultant DS.

7. A DS with the thresholds *W*_1_ and *W*_2_, is a DS such that for each node *F_i_ outside* the DS either *a. H*(*F_i_*) – (∑*_kj_*_:_*_kj_*_∈_*_DS_I*(*F_i_*, *F_ki_*) <*W*_1_ or *b*. *W*(*T_i_*) >*W*_2_

We used the following greedy algorithm to find the minimal dominant set with the thresholds *W*_1_ and *W*_2_:

*A.* Start with all the nodes as a DS.

*B.* At each stage, remove a node *F_j_* such that  is minimal.

*C.* Stop if .

8. Given the DS, the missing values in the target genome (*i.e.* unknown *F_k_*(*j*)) were reconstructed in the following manner:

*A.* Start with an initial guess of the missing values (*e.g.* the one suggested by the DS and/or the *T_i_* variables).

*B.* Based on this initial guess, infer all the labels of the co-evolutionary forest (with one of the algorithms for the ACE problem previously mentioned).

*C.* Change the labels of the missing values to improve the general parsimony score, given the labels at the ancestral states.

*D.* Repeat stages *B.* and *C.* till convergence (the change in the ACE score is lower than a certain threshold).

Note that we use the following approximation: *H*(*F_i_*|*F_k_*_1_, *F_k_*_2_,..) ≈ *H*(*F_i_*) – *I*(*F_i_*,* F_k_*_1_) – *I*(*F_i_*, *F_k_*_2_) – *…* Thus, it may be possible improve the accuracy (albeit increasing the running time) of the algorithm, by removing from the *DS* in each step the node *F_k_*, that minimizes . In addition, if one requires a range of sizes for dominant sets (and error rates) the thresholds *W*_1_, *W*_2_ may be altered.

#### Comparison of the different algorithms

In this section, we briefly report a comparison of the run times, and the quality of the solutions found by the aforementioned algorithms. The linear, integer, and quadratic programming were implemented in Matlab, using the commercial programming of TOMLAB optimization environment (http://tomopt.com/tomlab/). We used a Xeon 2.6GHz 64bit 2 cores x 4 cpu’s, with 4GB of memory. As can be seen (see Table 1), the linear programming archived a result that is optimal in terms of the quality of the solution (lowest and optimal parsimony score). The solution was similar to the one obtained by the ACE [27] (98.9 % of the inferred sites were identical). In addition, the running time of the FPT heuristic for solving the ACE [27] was shorter than all other algorithms, and the quality of the solutions found by this approach (with and without the greedy stage) is similar (though lightly higher) to the one obtained by the linear programming approach. The integer programming achieved the optimal solution (as the linear programming), but with a long run time. The integer quadratic programming and the min-cut heuristic, though theoretically interesting, were not practical for the large input we analyzed. The IQP failed due to memory problems, and the min-cut heuristic was not near convergence after a week of running.

**Table 1:** Comparison of the different algorithms for solving the *Ancestral co-evolution* problem.

Method	Network Score	Running Time
IP optimal	0.06	7.6 hr

LP rounded and not rounded	0.06	6 hr

FPT heuristic before greedy	0.063102751	1.83 hr

FPT heuristic	0.060550424	2 hr

IQP	–	fail (memory problems)

Min-cut	–	more than a week

#### The results of the linear programming

As mention in the previous section, the linear programming generally returns a solution ∈ [0, 1]. Thus, in general, the result found by the LP is a *lower bound* on the optimal (minimal) possible solution of the ACE problem. Interestingly, when we implemented the (linear programming) relaxation that was defined in the previous section, on the biological input, the values of *all* the variables that were assigned by the linear programming were ∈ {0, 1}. Thus, the linear programming found an *optimal* (and legal) solution for the problem. This result demonstrates, in accordance with subsection ’Some Computational Issues’, that in many practical cases the optimal solution can be found in polynomial time (for example by linear programming). In addition, this result shows that the ACE solutions found by the FPT heuristic in [26], are very near optimal (only 0.060550424/0.06 = 0.92% higher).

#### Demonstration of the algorithm for the *dominant co-evolutionary set* problem

We used the procedure for solving the *DCES* problem to analyze the genomes of six unicellular organisms. The first three bacteria were chosen according to their distance from the closest leaf in the phylogenetic tree: *P. aeruginosa*, *D. radiodurans*, and *E. coliOHE.* Among these three organisms, *E. coliOHE* has the closest leaf in the phylogenetic tree (other *E. coli* strains; 0.65% of the gene content is not similar) while *P. aeruginosa* has the lowest gene content similarity to its closest leaf in the phylogenetic tree (24% of the gene content is not similar). *D. radiodurans* has 18.3% non-similarity in gene content to its closest leaf in the phylogenetic tree. We analyzed three additional organisms: *S. cereυisiae* (an eukaryote; 2% dissimilarity to the closets leaf), *A. pernix* (an archaeon; 7% dissimilarity to the closest leaf), and *B. aphidicola* (an endosymbiont; 32% dissimilarity to the closest leaf).

The genome of each of these organisms was represented as a binary sequence, with 4873 entries (an entry for each gene families). The aim was to reconstruct parts of the genomes/sequences (*i.e.* determine the values, ’0’ or ’1’, of parts of the sequences) based on its remainder and the phylogenetic forest.

**Figure 5:**
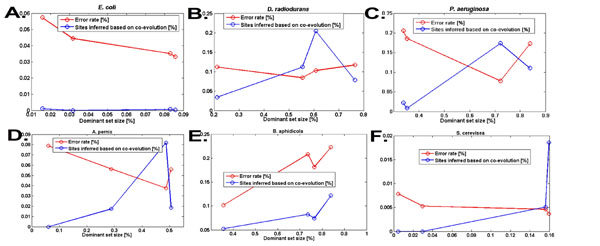
**Error-rate results of the DCES problem.** Implementation of the procedure for the *DCES* problem on six genomes: *E. coliOHE* (*A*.), *D*. *radiodurans* (*B*.), and *P. aeruginosa* (*C*.), *A. pernix* (*D.*), *B. aphidicola* (*E.*) *S. cerevisea* (*F.*). For each organism, the graph includes the error rate (red; % of the sites not in the DS were not reconstructed accurately based on the DS) and the % of sites that were reconstructed based on co-evolutionary relations (blue; *i.e.* their value cannot be inferred based on their evolutionary tree), for different sizes of the dominant set (% from the total number of proteins in the genome, x-axis).

**Figure 6:**
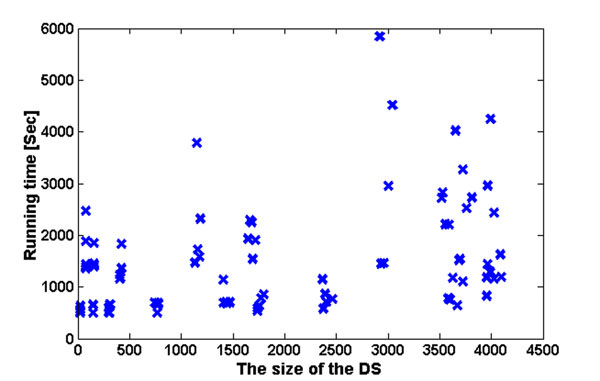
**Running time results of the DCES problem.** The figure includes the running time of 90 implementations of the DCES algorithm on the six analyzed organisms (15 samples for each organism), as a function of the size of the *DS*. The different sizes of the *DS* are a result of modifying the two thresholds (*W*_1_ and *W*_2_).

**Figure 7:**
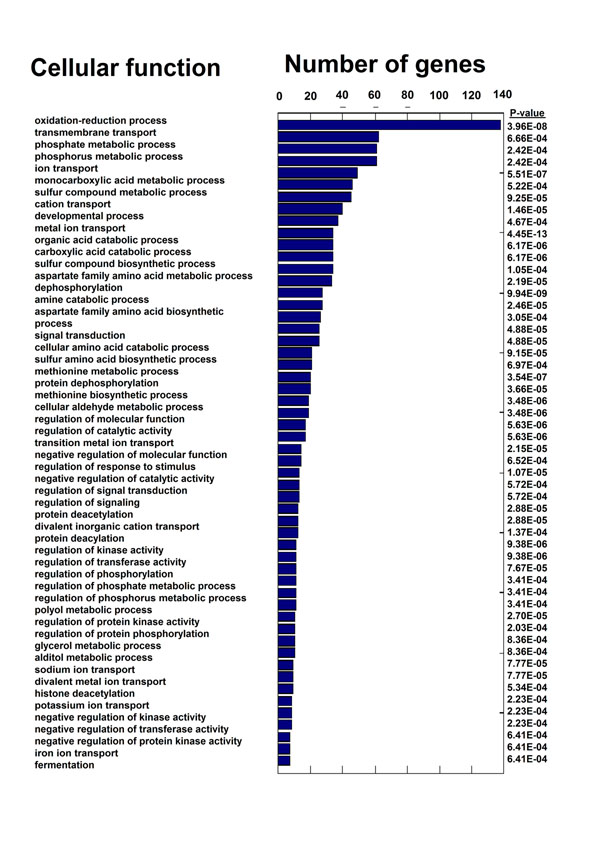
**The DCES problem: cellular function enrichment of the *DS* genes in S. cerevisea.** The figure includes cellular functions (biological process ontology) that are enriched in the *DS*, based on the genome of *S. cerevisea*.

We modified the thresholds *W*_1_, *W*_2_ to obtain various dominant set sizes, and computed the error rate when reconstructing the rest of the genome based on the *DS*. In addition, in each case, we computed the percentage of the reconstructed sites, that were inferred based on co-evolutionary information (*i.e.* not based on the *T_i_* variables; see the algorithm in the previous section). The results are depicted in Figures 5 – 7. The error-rate is represented as the percentage of the total number of reconstructed sites that we correctly inferred. The size of the *DS* is represented as the percentage of the sites (out of 4873), that were used to reconstruct the remaining sites.

##### Error rate

As can be seen, large portions of the genomes of organisms, such as *P. aeruginosa* and *D. radiodurans* (66% and 79% of the genome respectively), which do not have an evolutionary close neighbor in the co-evolutionary forest, can be reconstructed based on the rest of the corresponding genome, with a relatively low error rate (0.2 and 0.11 respectively). In addition, our results demonstrate that co-evolutionary information (and not only phylogenetic information) was used for the reconstruction of these genomes (up to 20% of the sites were inferred based on co-evolutionary information). It seems that co-evolutionary information is more important when there are no evolutionary close organisms in the co-evolutionary forest; for example, in the case of *E. coli* and *S. cerevisiae*, the fraction of sites that was inferred based on co-evolutionary data was relatively low. *B. aphidicola* is interesting as it undergoes a (’rare’) process of adaptation to a symbiotic lifestyle, where the gene set of the ancestor has been selectively reduced, so as to retain only those genes and pathways required for the new lifestyle [37,38]. The unique evolution of this endosymbiont challenged our approach, which is based on the statistic of the evolution of ’normal’ (non-endosymbiont) organisms. Indeed the error rate for this organism was slightly higher, but still surprisingly low (*e.g.* 0.1 for *DS* of size 35%).

Finally, the algorithm performed well for genomes from all three domains of life (error rate 0.04 and 0.005 for *A. pernix* and *S. cerevisiae* respectively).

### Running times

Figure 6 includes the running time of the procedure for solving the *DCES* problem as a function of the size of the *DS*. It includes the running time of 90 implementations of the DCES algorithm on the six analyzed organisms (15 samples for each organism), as a function of the size of the DS. The different sizes of the DS are a result of modifying the two thresholds (*W*_1_ and *W*_2_).

The typical running time for the analyzed phylogenetic forest is around 25 minutes (the range is between 8 and 97 minutes). Thus, the approach has practical running times.

As can be seen in the figure, the running time *usually* increases with the size of the *DS*. The running time when the *DS* includes less than 100 gene families is around 19 minutes, whilst the running time for cases with a *DS* larger than 3500 gene families is around 32 minutes.

### Biological analysis of the *DS* genes

We focused on *S. cerevisiae* aiming at understanding the properties of the *DS* genes. We decided to analyze *S. cerevisiae* as it is one of the most studied organisms in the analyzed dataset, with various public large scale measurements.

We began with studying the cellular function of the *DS* genes. To this end we performed functional enrichment analysis of the genes in the *DS* (Methods), based on the biological process ontology [39]. The results appear in Figure 7. As can be seen, the *DS* is mainly enriched with metabolic genes, genes related to transport, and genes related to various regulatory processes.

**Figure 8:**
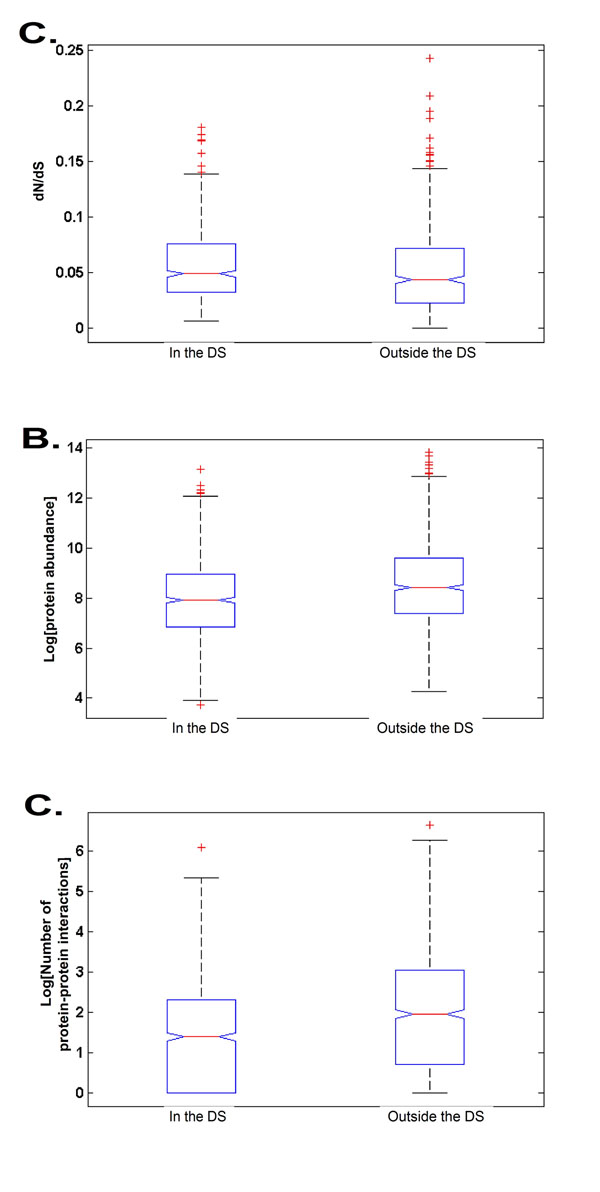
**The DCES problem: Properties of the *DS* genes in S. cerevisea.** Cellular properties of the *DS* genes in *S. cerevisea* demonstrate that the *DS* genes have higher *dN*/*dS* (*A*.), lower protein abundance (*B*.), and a lower degree in the PPI network (*C*.), compared to genes that are not in the *DS*.

We continued with a study of the cellular characteristics of the *DS* genes. In each case we compared the genes in the *DS* to the relevant set of genes that are outside the *DS* (Methods). At the first stage, we checked if the *dN*/*dS* (non-synonymous substitution rate divided by synonymous substitution rate) of genes in the *DS* is significantly different than the dN/dS of other genes. To this end, we used the data of [40]. We found the dN/dS of genes in the *DS* is significantly higher (0.0566 *vs.* 0.052; KS-test, *p* = 1.3913 ∗ 10^–5^; Figure 8A). Next, we checked if the Protein Abundance (PA) of genes in the *DS* is significantly different than the PA of other genes. To this end, we used the data of [41]. We found the PA of genes in the *DS* is significantly lower (1.2 ∗ 10^4^*vs.* 2.47 ∗ 10^4^; KS-test, *p* = 4.083 ∗ 10^–6^; Figure 8B). Next, we checked if the number of PP-interactions (PPI) of genes in the *DS* is significantly different than the number of PPI of other genes. To this end, we used the data of [26]. We found the number of PPI of the genes in the *DS* is significantly lower than the number of PPI of genes outside the *DS* (10.1 *vs.* 19.2; KS-test, *p* = 9.98 ∗ 10^–12^; Figure 8C).

The results presented in this section suggest that the *DS* genes include many metabolic genes, they have relatively high *dN*/*dS*, low protein abundance and low number of protein-protein interactions.

Genes with a high *dN*/*dS* tend to change rapidly between organisms, thus can be inferred less well based on other existing genomes. In addition, genes with a relatively low number of protein-protein interactions and protein abundance also tend to appear in a *DS*. Such genes have less functional constraints and can thus evolve faster. Furthermore, as such genes have less physical interactions and thus less co-evolutionary relations with other genes, their state can not be inferred by most of the other genes, and they should be added to the *DS*. The fact that most of the genes in the *DS* are metabolic and regulatory genes, demonstrates that these are the processes that tend to change among the analyzed organisms, supporting previous studies in the field [24,42-45].

## Conclusions

In this study we describe a few computational approaches for inferring genomes based on co-evolutionary relations. The algorithms described in this study are based on reductions to commonly employed approaches, such as linear programming (LP), quadratic programming (QP), and min-cut. As there are many free and commercial packages that solve LP and QP, the reductions describe in this study should be very useful in practice.

Furthermore, the current study also includes new results related to the computational complexity of the ACE problem. We report cases where an exact solution to the ACE problem can be found in polynomial time. As we demonstrate in the main text, such cases are common when analyzing biological data. Thus, in practice many times the optimal solution of the ACE problem can be found in a relatively short time. In addition, we describe a linear programming relaxation that returns a solution that can be used as a *lower bound* on the possible minimal solution. Thus, it can be used for estimating the quality of a legal solution found by the algorithms mentioned in this paper.

It is important to emphasize that the problem of finding a minimal and maximal cut can be solved more efficiently in graphs with certain properties. Thus, the approach min/max-cut reduction, suggested in this study, may be useful in such cases. For example, it is known that the max-cut problem can be solved in polynomial time in planar graphs [46]. Thus, if the co-evolutionary forest is planer, the ACE with only *red* edges can also be solved in polynomial time.

Finally, we formally describe for the first time strategies for 1) inferring a genome based on a portion of it, and 2) finding a part (subset of the proteins) of a target genome such that it will be possible to reliably reconstruct the rest of the target genome base on this subset. Thus, by using this strategy one can sequence only a section of a genome of interest, and infer its entire gene content. This approach can be generalized to deal with the inference of cellular networks (*e.g.* metabolic networks and protein-protein interaction networks). In these cases, the input includes a target organism with a partial cellular network and the cellular networks in other organisms; the aim is to infer the rest of the cellular network of the target organism. One of the major differences in the case of this generalization, is the fact that *both* the nodes and the edges of the network need be inferred.

## Methods

### The analyzed co-evolutionary forest

The evolutionary tree, the labeling of the leaves, and the co-evolutionary information were downloaded from [26]. This data includes the gene content (4873 gene families) of 95 unicellular organisms (bacteria, archaea, and eukaryotes). The classification to gene families was based on the COG database [36,47]. See [26] for more details regarding the input.

### The co-evolutionary edges

**Figure 9:**
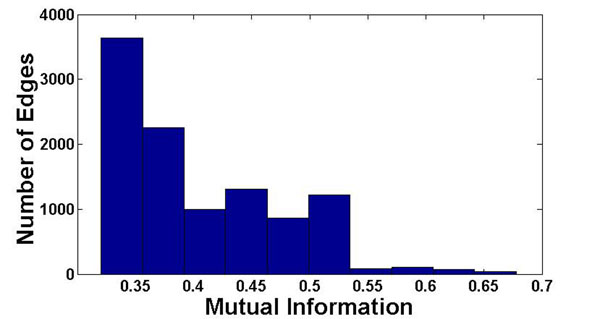
**The distribution of mutual information on the co-evolutionary edges.** The distribution of mutual information scores for the co-evolutionary edges used in this study.

We used the co-evolutionary data from [26]. These data include pairs of proteins that exhibit various physical and functional interactions. We ranked pairs of proteins (co-evolutionary edges) according to the empirical mutual information between their gene content vectors. For two proteins *x*, and *y* let *p*(*x*), *p*(*y*), be the empirical distribution of the state (’1’ or ’0’; appear or disappear in the genome) of the proteins over the analyzed organisms, and let *p*(*x*, *y*) be the joint empirical distribution of the protein pair. The corresponding empirical mutual information is *I*(*x*, *y*) = *Σp*(*x*, *y*) *· log*(*p*(*x*, *y*)/*p*(*x*) *· p*(*y*)). Higher mutual information corresponds to stronger co-evolution. The final co-evolutionary forest included 10, 576 edges (Figure 9). The weight table of a pair of COGs included the *–log*(·) of the joint empirical distribution of the two COG.

To estimate the number of red and green edges in the co-evolutionary forest we computed the KL distance between the weight table of each edge and the weight tables of the green and red edges that were defined in subsection ’Some Computational Issues’. The empirical KL distance is defined as *KL*(*x*||*y*) = Σ*p*(*x*) *· log*(*p*(*x*)/*p*(*y*)). We found that 142 of the edges were red (KL distance to the red weight table is lower) and the rest of them were green (KL distance to the green weight table is lower).

The red edges relates to pairs of COG that tend to mutually exclude each other (if a gene of one of the COG appear in the organism the second usually does not appear in this organism). For example the edge between *COG*1467 (Eukaryotic-type DNA primase, catalytic (small) subunit) and *COG*2812 (predicted type IV restriction endonuclease) is red. The first one tend to appear in archaea eukaryotes and the second in bacteria.

### GO enrichment analysis and analysis of the cellular features of DS genes

In all the GO enrichment analyzes, the set of *S. cerevisiae* genes that was mapped to the *DS* COGs was compared to the *S. cerevisiae* genes that have a mapping to COGs as a background. Similarly, the PA, PPI, and dN/dS of the set of *S. cerevisiae* genes that was mapped to the *DS* COGs was compared to the features of the *S. cerevisiae* genes that have mappings to COGs.

## Competing interests

The authors declare that they have no competing interests.

## Authors' contributions

HB and TT participated in the design and execution of the study; HB and TT analyzed the results; TT participated in the preparation of this manuscript. HB and TT read and approved the final manuscript.
